# Anomalies of Typhoid Fever

**Published:** 1895-10

**Authors:** C. J. Forbes

**Affiliations:** Round Rock, Texas


For Texas Medical Journal.

### ANOMALIES OF TYPHOID FEVER.

BY C. J. FORBES, M, D., ROUND ROCK, TEXAS.

Read at the Austin District Medical Society.

MR. PRESIDENT, it is my purpose to invite the attention of this body to some anomalies noticed in two or three cases of typhoid fever.

It is impossible for a physician to begin practice with a true conception of all diseases. In fact, it may be doubted, whether the young doctor of ordinary advantages, has the true conception of any disease.

Many illusions necesarily exist. Among the first of these to be dispelled by practical experience is the one expecting to find typical cases of a given disease. He does not practice long before he finds, contrary to expectation, that the atypical is the rule. Even in well-known typhoid fever the anomalous manifestations are so numerous that his experience must be very large before he has met them all. If every disease had one or more pathognomonic symptoms present and continuing through every case, diagnosis would be easy. Most diseases present, occasionally, unusual features, that may perplex and lead us astray. Typhoid fever is a very complex disease and presents a very diversified symptomatology, which has led to many clinical errors.

A majority of cases of one epidemic may be typical and those of another atypical. Again, the symptoms of this disease seem to be greatly modified by such influences as climate, altitude and concurring diseases. In the South its symptoms are modified and changed by malarial complications, while typhoid occurring in the by altitudes of the Rocky Mountains presents, seemingly, from what I have read, quite a different series of symptoms.

In reference to the malarial complication, Prof. Osler says: "I confess myself unable to differentiate certain cases of malarial remittent from typhoid fever without the blood examination." No wonder, then, that there should be so much doubt and dispute among the great mass of physicians as to the existence or non-existence of typho-malarial fever. Among the physicians of the Rocky Mountain regions there seems to exist a difference of opinion as to the nature of their Rocky Mountain fever. The last edition of Dunglison's dictionary defines this as "a form of typho-malarial fever met with in high altitudes, as in the Rocky Mountains."

In the *Medical News*, of April 8th, 1893, is an article, by Dr. Work, of Pueblo, on Mountain Fever. In this article he gives an account of thirty-two cases of mountain fever, admitted to hospital in Colorado Springs in the summer and fall of 1890. In eighteen of these the rose colored spots of typhoid were seen; in six they were absent, and in eight their presence or absence is not stated. Five cases of the thirty-two terminated fatally, and the post mortem examinations showed in each case the lesions of typhoid fever.

He concludes that the great majority of cases of continued fever occurring in the Rocky Mountains are typhoid, and should be so classed.

The object of the foregoing is to call attention to the fact that the great diversity of symptoms occurring in typhoid fever causes a great diversity of opinion, but post mortems show that a vast majority of cases of severe continued fever are true typhoid. But to come more directly to my subject. In October, 1892, I was called to see a boy sick with fever. This was a family of Swedes, and consisted of parents and three sons, varying in age from eleven to sixteen years. During the fall the father and the boys had the fever. The oldest boy was the second to take sick. In the fourth week of the fever he was seized with a severe pain in the right iliac fossa. This was soon followed by a general peritonitis, of which he soon died. To my mind this was a clear case of perforation. The first boy sick had a very severe attack, but recovered; the third had a mild case. On the first of November the father had an attack of fever, which yielded at once to a mercurial purge and quinine. Although he had no fever he did not seem to be entirely well. During the last days of November he had a similar attack, and was treated the same way, with the same result.

He continued free of fever and able to do some work until December 10th, when he was taken with diarrhœa, and fever returned. I again gave quinine, and the fever disappeared at once, but the diarrhœa persisted for some time, in spite of active treatment. From December 11th to the 21st he had no fever, but his temperature was frequently subnormal. December 17th it was 96.5°. The family had a thermometer, and tested his temperature frequently each day. I have no record of December 18th, but the 19th and 20th the temperature was subnormal. For about four days preceding his death, the temperature arose to 102. It ran up at one time to 106, but 5 grs. of antikamnia very promptly reduced it to 100°. December 17th his bowels became slightly tympanitic, and this condition increased up to his death, which occurred December 25th.

He never complained of pain at any time, although repeatedly questioned on this point. Ten hours after his death, I held a post mortem, assisted by Drs. Holloway and Norris. We found three perforations, situated from ten to sixteen inches above the cæcum. One of them was a slit in the bottom of a large ulcer.

This slit was made larger by handling. The other two were round openings at the bottom of small ulcers.

One peculiarity of this case, is the absence of pain after perforation. No fecal matter had escaped into the abdominal cavity, and this doubtless is the explanation. But the anomaly to which I wish particularly to direct your attention, is the absence of fever during the whole of the sickness. This man was 46 years of age, and I am aware that typhoid fever does not range so high in aged subjects, but it is certainly unusual for them to have no fever.

In looking through medical literature at my command for similar cases, I find one recorded by Dr. Taylor, of Rhode Island. His patient was a young married woman with atresia of vagina. To this abnormality he attributed her sickness, as she had scarcely any fever. But the post mortem showed the characteristic ulcers of typhoid fever, and no inflammation of the genital organs.

In the *Medical News* of May 13th, 1893, the editor refers to four cases reported in *The Practitioner* by another physician. These four cases presented the classical signs and symptoms of enteric fever, with the exception of pyrexia.

Osler says: "An afebrile typhoid fever is recognized by authors. Lerbermeister says such cases were not uncommon at Basle." He adds, "I have no personal knowledge of such cases."

On October 17, 1893, I was called to see a man who had been confined to his bed with fever for eleven days. This old man—also a Swede—-was in his 67th year. Up to that time, he had had no medical attention. His tongue was dry, and heavily coated; the teeth were covered with sordes. There was a marked subsultus, not only of the tongue, but of all the limbs. A muttering delirium was present, except when aroused. No rose colored spots were present. On examining the abdomen, I found it absolutely flat. Not only the spinal column, but also the abdominal aorta, could be easily outlined.

This condition, and his age, were the anomalies of this case. The fever ranged from 100 to 103. So far as I could learn, there had never been any cases of typhoid fever on this place, nor was it prevailing anywhere in the country at that time. He died five days after I saw him. If this was not a case of typhoid fever, I am at a loss what to call it.

I do not remember noticing anything in reference to absence of tympanites in this disease, except in a review of A. Strumpell's

"Practice of Medicine," in the *Therapeutic Gazette*. The reviewer says Strumpell does not consider tympanites a common condition in this disease.

